# Supplementary material for: Unexpected cell type-dependent effects of autophagy on polyglutamine aggregation revealed by natural genetic variation in C. elegans
Source: BMC Biol. 2020 Feb 24;18:18. doi: 10.1186/s12915-020-0750-5 (PMC7038566; doi:10.1186/s12915-020-0750-5)
Supplement: Supplementary file 1 — Additional file 1: Figure S1. Schematic of the drxlR1 interval and SNPs used for mapping. (A) Red: the 1.4 Mb genomic region on chromosome I, containing the DR1350-derived intervals, in the RIL2-derived drxlR1;Q40 strain and the four remaining high aggregation RILs (RIL12, RIL12(2), RIL18 and RIL15); orange: the Bristol background. Punctate lines delineate the narrowed 326 Kb interval containing the candidate genes tested by RNAi. Diamonds: SNPs used to test for the presence of the interval; SNP 6b (ChrI:1,972,719 (WBVar00017376)) is Bristol-derived in drxIR1;Q40 and RIL15 animals. Locations of egl-30, moag-4 and the incompatibility locus zeel-1/peel-1are also indicated. The coordinates here correspond to the WormBase release WS270 [131]. (B) WormBase names and chromosomal locations of SNPs marked with diamonds in A. [file 12915_2020_750_MOESM1_ESM.pptx]

## Slide 1
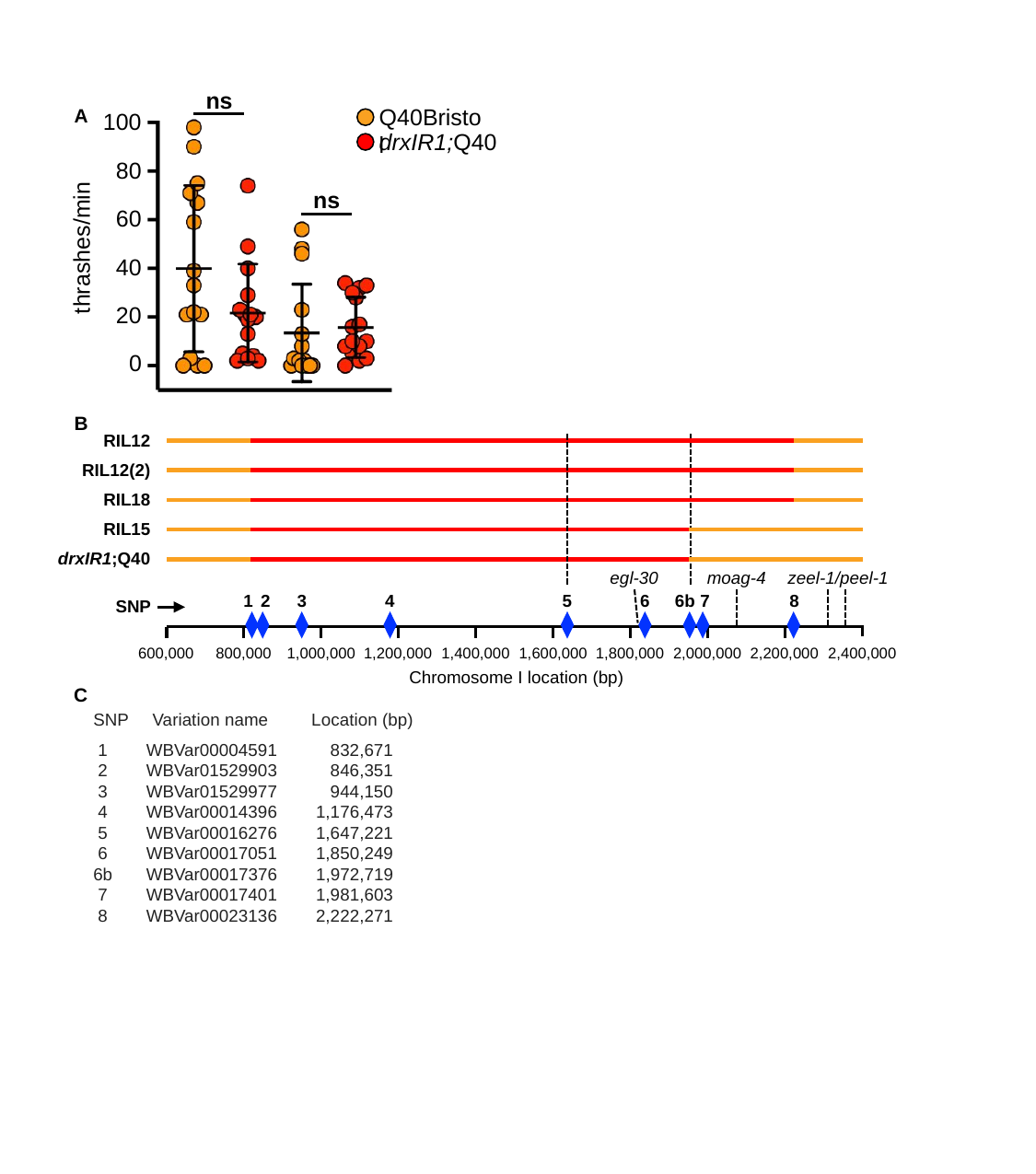

ns
100
80
ns
60
thrashes/min
40
20
0
Q40Bristol
A
drxIR1;Q40
B
RIL12
RIL12(2)
RIL18
RIL15
drxIR1;Q40
egl-30
moag-4
zeel-1/peel-1
1
2
3
4
5
6
6b
7
8
SNP
600,000
800,000
1,000,000
1,200,000
1,400,000
1,600,000
1,800,000
2,000,000
2,200,000
2,400,000
Chromosome I location (bp)
C
SNP Variation name Location (bp)
 1 WBVar00004591 832,671
 2 WBVar01529903 846,351
 3 WBVar01529977 944,150
 4 WBVar00014396 1,176,473
 5 WBVar00016276 1,647,221
 6 WBVar00017051 1,850,249
6b WBVar00017376 1,972,719
 7 WBVar00017401 1,981,603
 8 WBVar00023136 2,222,271
