# Supplementary figures and images for: Unexpected cell type-dependent effects of autophagy on polyglutamine aggregation revealed by natural genetic variation in C. elegans
Source: BMC Biol. 2020 Feb 24;18:18. doi: 10.1186/s12915-020-0750-5 (PMC7038566; doi:10.1186/s12915-020-0750-5)

## Slide 1
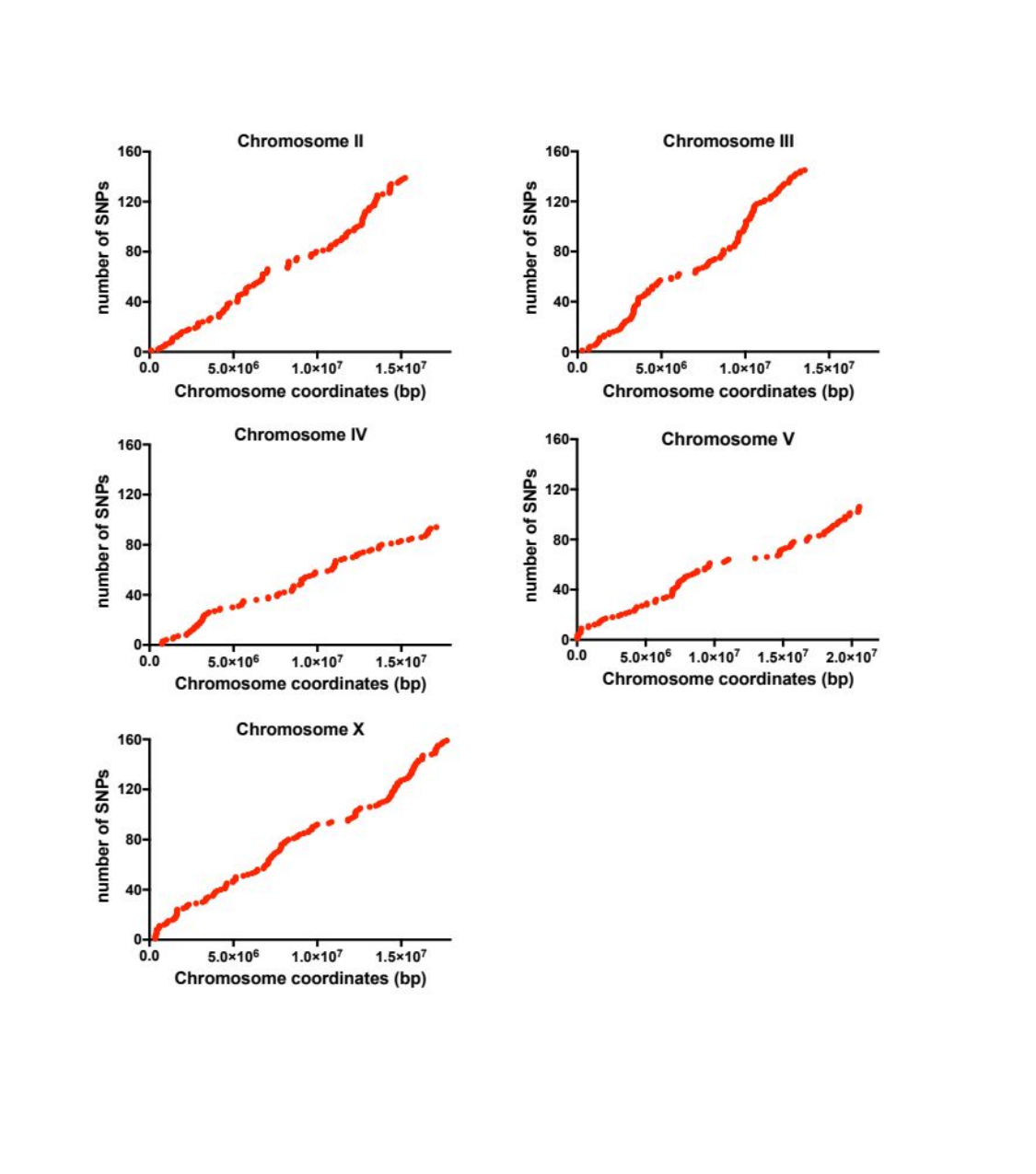

Supplement: Supplementary file 2 — Additional file 2: Figure S2. Cumulative distribution of unique SNPs across remaining chromosomes. chromosomes II through X in the drxlR1;Q40 strain accumulated up to 160 unique SNPs each. Shown are SNPs remaining after subtraction of the variants present in Q40Bristol strain, and of variants in the Hawaiian isolate that does not exhibit increased polyQ40 aggregation. [file 12915_2020_750_MOESM2_ESM.pptx]

## Slide 1
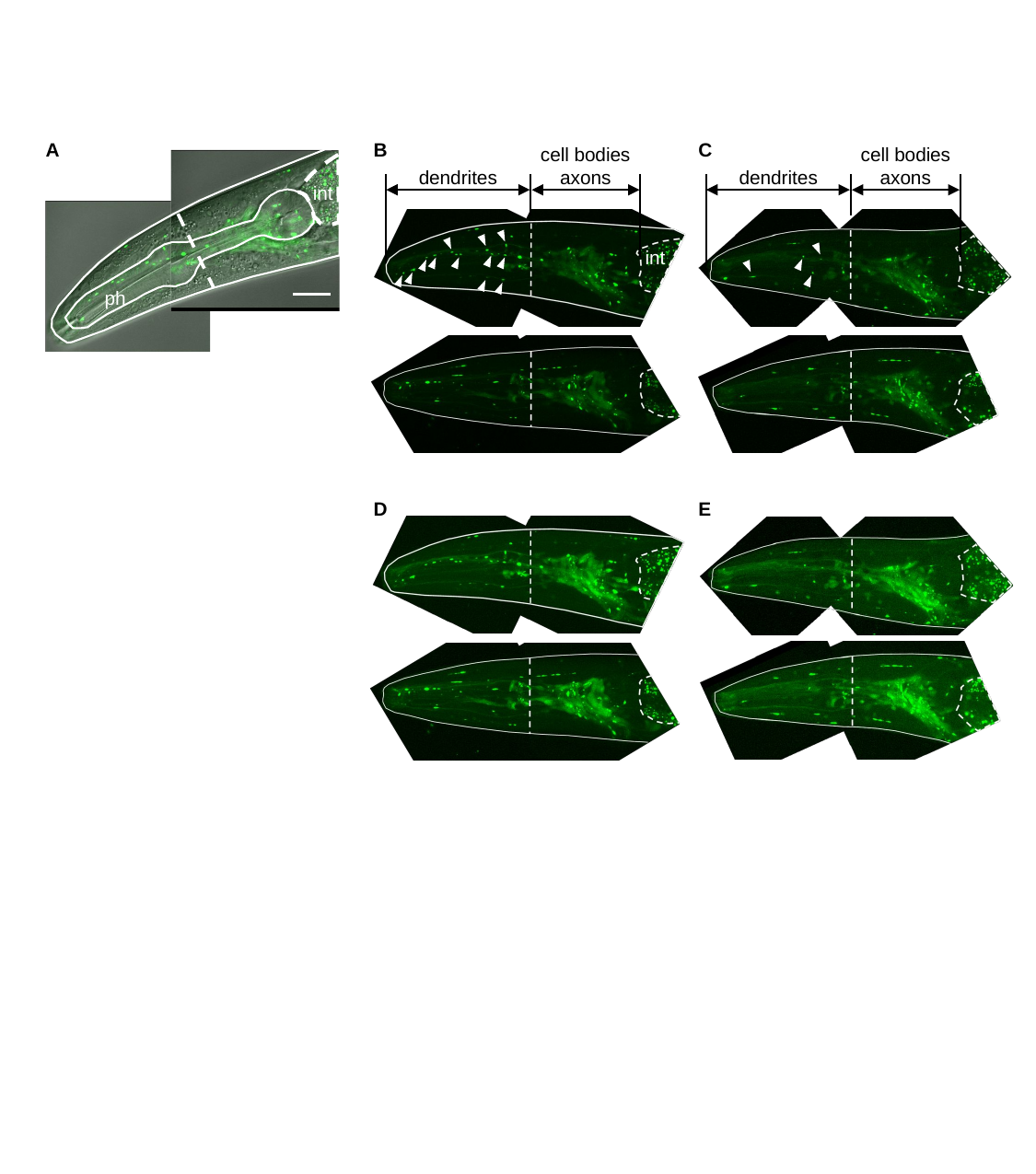

A
int
ph
B
cell bodies
axons
dendrites
int
C
cell bodies
axons
dendrites
D
E

Supplement: Supplementary file 4 — Additional file 4: Figure S4. The drxIR1 interval decreases accumulation of polyQ67 aggregates in the neurites. (A) Outline of the head of an individual animal, with intestine (int) and pharynx (ph) indicated. Anterior to the left, dorsal up. Green shows projection of Q67n::CFP fluorescence signal overlaid on the transmitted light image. Punctate line in the middle immediately below the anterior pharyngeal bulb separates the dendritic region where aggregation was scored (anterior to the line) from the area with many neuronal cell bodies and the axonal bundle. The scored region contains mainly dendrites of sensory neurons, with some interneuron processes and some cell bodies and/or neurites of other types of neurons (https://www.wormatlas.org/neuronsandcircuits.html). Scale bar is 20 μm. Same animal as in panel B, top. (B) Confocal images of heads of two Q67n::CFP animals in Bristol background, rotated into horizontal positions. Regions scored in Fig 5f are anterior (to the left) to the punctate line (marked as dendrites). Examples of aggregates within the neurites are indicated by arrowheads. (C) Confocal images of drxIR1;Q67n::CFP animals. Labeling as in panel B. Areas marked as cell bodies and axons did not exhibit decreased aggregation in drxIR1 background relative to Bristol (compare to the same area in panel B). (D, E) Same images as in panels B and C, respectively, shown with enhanced fluorescence. [file 12915_2020_750_MOESM4_ESM.pptx]
