# Supplementary material for: Unexpected cell type-dependent effects of autophagy on polyglutamine aggregation revealed by natural genetic variation in C. elegans
Source: BMC Biol. 2020 Feb 24;18:18. doi: 10.1186/s12915-020-0750-5 (PMC7038566; doi:10.1186/s12915-020-0750-5)
Supplement: Supplementary file 3 — Additional file 3: Figure S3. Controls for the basal proteostasis effects of the drxIR1 locus. (A) The UbG76V::Dendra2 proteasome reporter is sensitive to decreased proteasome levels. Knockdown of a proteasome subunit rpn-6.1, via RNAi, increased the average intensity of the Dendra2 compared to control treatment. Images were taken and quantified as in Fig. 2a. Data are mean ± SD. Data were analyzed by unpaired t-test, two-tailed, *P=0.0244. (B) Stereomicrographs of young adult animals after treatment with control or moag-4 RNAi. moag-4 RNAi decreased aggregation in both backgrounds, but preserved the increased aggregation drxIR1;Q40 animals relative to Q40Bristol. [file 12915_2020_750_MOESM3_ESM.pptx]

## Slide 1
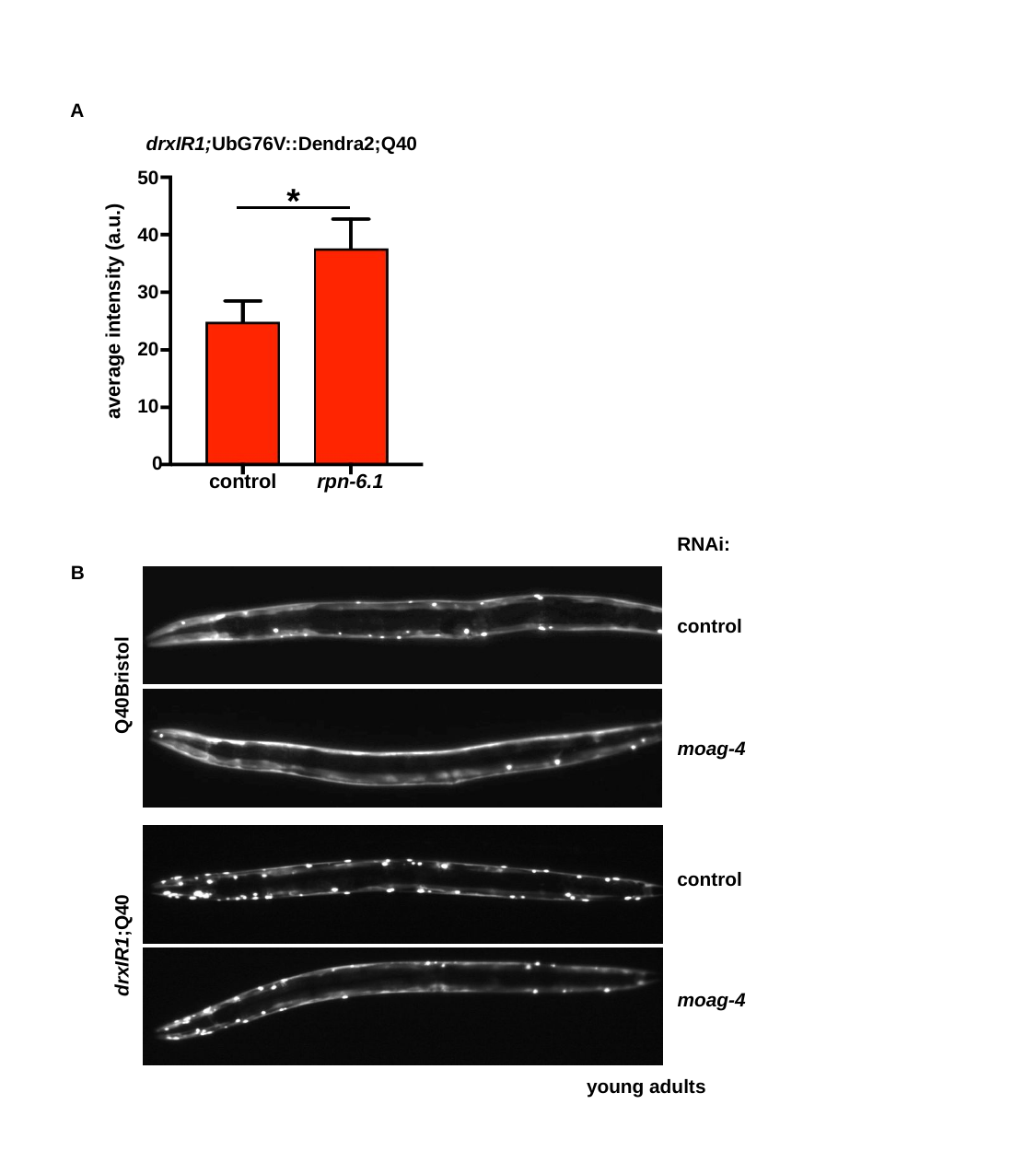

A
drxIR1;UbG76V::Dendra2;Q40
50
*
40
30
average intensity (a.u.)
20
10
0
control
rpn-6.1
RNAi:
B
control
Q40Bristol
moag-4
control
drxIR1;Q40
moag-4
young adults
