# Supplementary material for: Unexpected cell type-dependent effects of autophagy on polyglutamine aggregation revealed by natural genetic variation in C. elegans
Source: BMC Biol. 2020 Feb 24;18:18. doi: 10.1186/s12915-020-0750-5 (PMC7038566; doi:10.1186/s12915-020-0750-5)
Supplement: Supplementary file 5 — Additional file 5: Table S1. Loss-of-function analysis for the RIL2-like head aggregation phenotype. sDP2 free duplication covers most of the left arm of chromosome I, extending through dpy-5 marker but not through unc-13. drxIR1;Q40 animals were crossed with KR292 [him-1(h55);dpy-5(e61);unc-13(e450)I; sDp2(I;f)], F1 progeny that either did (based on segregation of unc non-dpy phenotype among their progeny) or did not inherit the sDp2 duplication were singled, and their F2 progeny scored for the increased ratio of head to body aggregation (RIL2-like) and the dumpy phenotypes. The RIL2-like phenotype behaved genetically as did the known loss-of-function dpy-5(e61) allele. [file 12915_2020_750_MOESM5_ESM.docx]

| **Suppl. Table 1 Loss of function analysis for the increased susceptibility of head muscles to aggregation** | | | | | |
| --- | --- | --- | --- | --- | --- |
| **F1 animals that *did not* inherit sDp2** | | | | | |
| # of F1 animals | # of F2 animals scored | # RIL2-like  F2 animals | # *dpy*  F2 animals | % RIL2-like  F2 animals | % *dpy*  F2 animals |
| 22 | 4533 | 1041 | 1109 | 23 | 24.5 |
|  | | | | | |
| **F1 animals that *did* inherit sDp2** | | | | | |
| # of F1 animals | # of F2 animals scored | # of RIL2-like  F2 animals | # of *dpy*  F2 animals | %RIL2-like  F2 animals | % *dpy*  F2 animals |
| 5 | 707 | 91 | 91 | 12.8 | 12.8 |
