# Supplementary material for: Unexpected cell type-dependent effects of autophagy on polyglutamine aggregation revealed by natural genetic variation in C. elegans
Source: BMC Biol. 2020 Feb 24;18:18. doi: 10.1186/s12915-020-0750-5 (PMC7038566; doi:10.1186/s12915-020-0750-5)
Supplement: Supplementary file 6 — Additional file 6: Table S2. Candidate genes tested by RNAi. 24 candidate genes present in the target 326 Kb of drxIR1 interval (between SNPs 5 and 6b (Additional file 1: Fig. S1)) are indicated in color. Genes were defined as candidates based on the SnpEff annotations (see Methods and Additional file: Data File 1). egl-30 was excluded based on genetic crosses. Genes in purple were targeted by clones from the Ahringer RNAi library. RNAi targeting constructs for genes in red were prepared in this work. [file 12915_2020_750_MOESM6_ESM.docx]

| **Suppl. Table 2 Genes located in the 350Kb interval on the left arm of Chromosome I of *drxIR1* animals** | | |
| --- | --- | --- |
| **Gene** | **Candidate** | **Function/homology/phenotype** |
| ***mab-20*** | + | Semaphorin-2A ortholog; is required for proper epidermal morphogenesis and axon guidance |
| ***Y71G12B.18*** | + |  |
| ***Y71G12B.33*** | + |  |
| ***Y71G12B.17*** | + | An ortholog of human PITPNB and PITPNA; may have phospholipid transporter activity |
| ***Y71G12B.23*** | + | An ortholog of human MMD and MMD2; may have heme-copper terminal oxidase activity |
| ***Y71G12B.35*** | + |  |
| ***drag-1*** | + | A membrane associated protein that functions as a co-receptor in the the Sma/Mab pathway |
| ***Y71G12B.31*** | + | An ortholog of human PTPN7; may have protein tyrosine phosphatase activity |
| ***ubc-3*** | + | E2 ubiquitin-conjugating enzyme |
| ***atg-5*** | + | An ortholog of the autophagic budding yeast protein Atg5p, and of human ATG5 |
| ***tln-1*** | + | Talin; a cytoskeleton protein |
| ***mppa-1*** | + | Mitochondrial processing peptidase alpha |
| ***lin-65*** | + | Acts with B class SynMuv genes to repress vulval induction, and is required for fertility. Component of the mitochondrial unfolded protein response |
| ***Y71G12B.25*** | + | An ortholog of human MFSD10 |
| ***Y71G12B.5*** | + |  |
| ***pghm-1*** | + | Peptidylglycine alpha-hydroxylating monooxygenase |
| ***chaf-2*** | + | Chromatin assembly factor |
| ***C53H9.3*** | + |  |
| ***egl-30*** | - | An ortholog of the heterotrimeric G protein alpha subunit Gq; affects viability, locomotion, egg laying, synaptic transmission, and pharyngeal pumping |
| ***tag-96*** | + | A galactokinase that is a member of the GHMP family of kinases |
| ***tub-2*** | + | Tubby-related |
| ***Y71G12A.4*** | + | An ortholog of human ABHD17A and ABHD17C |
| ***trpp-10*** | + | Transport protein particle |
| ***Y51F10.4*** | + | An ortholog of human SLC38A10 |
| ***spe-48*** | + | Spermatogenesis defective |
