# Supplementary material for: Unexpected cell type-dependent effects of autophagy on polyglutamine aggregation revealed by natural genetic variation in C. elegans
Source: BMC Biol. 2020 Feb 24;18:18. doi: 10.1186/s12915-020-0750-5 (PMC7038566; doi:10.1186/s12915-020-0750-5)
Supplement: Supplementary file 7 — Additional file 7: Table S3. Primers used for genotyping the drxIR1 locus and for qPCR analysis of atg-5 expression. [file 12915_2020_750_MOESM7_ESM.docx]

| **Suppl. Table 3 Primers used for genotyping the *drxIR1* locus and for qPCR analysis of *atg-5* expression** | |
| --- | --- |
| **Primer name** | **Primer sequence 5’-3’** |
| drxIR1 fwd. | AAGCCCCGCCGAGTTAAAACCG |
| drxIR1 rev. | TGGCGACCGAGTGTAGCATCGTG |
| atg-5 fwd. | CTGGCGGAACTCACGGAG |
| atg-5 rev. | CGCTTGATCGTAGATCAC |
| tbg-1 fwd. | CCTGTTGTCGATCCAAATGA |
| tbg-1 rev. | AACCCGAGAAGCAGTTGAAA |
