# Supplementary material for: Unexpected cell type-dependent effects of autophagy on polyglutamine aggregation revealed by natural genetic variation in C. elegans
Source: BMC Biol. 2020 Feb 24;18:18. doi: 10.1186/s12915-020-0750-5 (PMC7038566; doi:10.1186/s12915-020-0750-5)
Supplement: Supplementary file 8 — Additional file 8: Table S4. Primers used for generating RNAi clones. [file 12915_2020_750_MOESM8_ESM.docx]

| **Suppl. Table 4 Primers used for generating RNAi clones** | |
| --- | --- |
| **Primer name** | **Primer sequence 5’-3’** |
| mab-20_HindIII Up | GACGAAGCTTAACTCGGCCACCTATTGAAC |
| mab-20_NcoI Down | AGTACCATGGAGCAACTGAGAATTGGGAGAC |
| Y71G12B.18_HindIII Up | GACGAAGCTTGGTAGCGGATGACTGATGTTAG |
| Y71G12B.18_NcoI Down | ACTTCCATGGAGCTCCTTTACAAACAGGTAGG |
| Y71G12B.33_NcoI fwd. | AGTACCATGGACGCTTGCCATTCGAAATAAAC |
| Y71G12B.33_HindIII rev. | TCATAAGCTTCATGCCTCCTCCCATTCATC |
| Y71G12B.23_HindIII Up | ACTGAAGCTTGATCGGTATAGGACCCACAATTC |
| Y71G12B.23_NcoI Down | GACTCCATGGGCCGCAATGAACAGGTAAATG |
| Y71G12B.35_NcoI fwd. | TAGTCCATGGTTGAGTCGAAGGAGCCAAAG |
| Y71G12B.35_HindIII rev. | TACGAAGCTTTCAAACGGAGCAAATTGAGAAAG |
| drag-1_HindIII Up | CGTCAAGCTTCTGGAACAGAATAAGTTG |
| drag-1_NcoI Down | AGCACCATGGCCCATCACATCGTGTCGT |
| Y71G12B.31_NcoI fwd. | TACTCCATGGCTACTTCTTCTGATGGTAGTTCCTC |
| Y71G12B.31_HindIII rev. | TACTAAGCCTTCCGTACTGGTGTGTTCATCTG |
| ubc-3_NcoI fwd. | TACTCCATGGCAGGTGGAAGAGTCGAAGAAAG |
| ubc-3_HindIII rev. | TACTAAGCTTCGTCATAGTCACACCCGAAATC |
| tln-1_AB_SalI Up | GCATGTCGACCGTCTCGAACAAGACTGTACTC |
| tln-1_AB_BglII Down | ACTGAGATCTAGTAGAGCGCGTTTGTATGG |
| Y71G12B.25_NcoI fwd. | ACTACCATGGCAAGGCAATTTGAGTGTTGGAG |
| Y71G12B.25_HindIII rev. | TACTAAGCTTTCAATGGTCCAATGGCTCAC |
| pghm-1_NcoI fwd. | TACTCCATGGAAACAGGAACCCGATGAGAC |
| pghm-1_HindIII rev. | ATGCAAGCTTGGCTTCACAAACAGATCAACAG |
| C53H9.3_NcoI fwd. | ATCTCCATGGCAATCCCTTAATTCTTTCCAGA |
| C53H9.3_HindIII rev. | ATCTAAGCTTCTTGATCCTTACAACGGGTAG |
| M01D7.4_BglII fwd. (tag-96 fwd.) | TACTAGATCTTCGCCTCAAAGAAATTCAAACC |
| M01D7.4_SalI rev. (tag-96 rev.) | TGATGTCGACTGCGATGACTCTCGTTCATTAG |
| Y71G12A.3_NcoI fwd. (tub-2 fwd.) | ATCACCATGGGTTTGTTAAGCACTGCCCTAT |
| Y71G12A.3_HindIII rev. (tub-2 rev.) | TACTAAGCTTTGGCTACTGAAGCGCTAGTG |
| Y51F10.4_BglII fwd. | TACTAGATCTAGGAGAGGCAGCATCAGAAG |
| Y51F10.4_SalI rev. | TACTGTCGACGCTTAAAGCATTTCTGGCAAC |
| Y51F10.10_NcoI fwd. (spe-48 fwd.) | TACTCCATGGCGTTCAGTAGACACAAAGGAAGAC |
| Y51F10.10_HindIII rev. (spe-48 rev.) | TACTAAGCTTGATGAAACGTTGCCGTTCTTG |
