# Supplementary material for: Unexpected cell type-dependent effects of autophagy on polyglutamine aggregation revealed by natural genetic variation in C. elegans
Source: BMC Biol. 2020 Feb 24;18:18. doi: 10.1186/s12915-020-0750-5 (PMC7038566; doi:10.1186/s12915-020-0750-5)
Supplement: Supplementary file 9 — Additional file 9: Data File 1. List of genes in the drxIR1 interval with potentially significant SNPs generated by the SnpEff tool. The nucleotide positions correspond to the N2(Bristol) genome assembly from WormBase release WS220 [131], available in the UCSC Genome Browser as ce10. The presence of human orthologs is according to [132]. [file 12915_2020_750_MOESM9_ESM.pdf]

Data File 1

# Chr	Position	Reference	Change	Type	Homoz	Quality	Coverage	Gene_name	Bio_type	Effect	old/new AA	Old/New codon	Codon Degen	Human orthologs
I	1647223	C	G	SNP	Hom	80.72	3	mab-20	Semaphorin	NON_SYNONYMOUS_CODING	V/L	Gtc/Ctc	0	+
I	1648246	T	C	SNP	Hom	404.04	11	mab-20		NON_SYNONYMOUS_CODING	I/V	Atc/Gtc	0	
I	1649294	A	C	SNP	Hom	174.42	7	mab-20		UTR_3_PRIME: 41 bases from CDS				
I	1649298	C	T	SNP	Hom	214.39	8	mab-20		UTR_3_PRIME: 37 bases from CDS				
I	1649399	A	G	SNP	Hom	77.6	3	mab-20		NON_SYNONYMOUS_CODING	F/S	tTt/tCt	0	
I	1653522	C	A	SNP	Hom	486.38	13	Y71G12B.18	protein_coding	NON_SYNONYMOUS_CODING	H/Q	caC/caA	1	
I	1660980	A	T	SNP	Hom	446.38	12	Y71G12B.33	protein_coding	NON_SYNONYMOUS_CODING	L/M	Ttg/Atg	1	
I	1667646	T	C	SNP	Hom	443.35	12	Y71G12B.17	protein_coding	UTR_5_PRIME: 84 bases from TSS				
I	1670189	*		INS	Hom	124.21	8	Y71G12B.23	protein_coding	UTR_3_PRIME: 21 bases from CDS				+
I	1673156	C	A	SNP	Hom	348.73	11	Y71G12B.35	protein_coding	NON_SYNONYMOUS_CODING	G/V	gGa/gTa	0	
I	1680477	G	A	SNP	Hom	420.86	12	drag-1	Sma/Mab regulator	UTR_5_PRIME: 268 bases from TSS				+
I	1682968	T	C	SNP	Hom	324.04	9	drag-1		NON_SYNONYMOUS_CODING	Y/H	Tat/Cat	0	+
I	1687806	C	T	SNP	Hom	287.08	8	Y71G12B.31	protein_coding	NON_SYNONYMOUS_CODING	R/K	aGa/aAa	0	
I	1698160	C	T	SNP	Hom	247.11	7	ubc-3	E2	UTR_5_PRIME: 193 bases from TSS				+
I	1701041	*		INS	Hom	406.03	15	ubc-3		UTR_3_PRIME: 252 bases from CDS				
I	1715498	G	T	SNP	Hom	324.04	9	**atg-5**	autophagy	UTR_3_PRIME: 101 bases from CDS				+
I	1715525	*		DEL	Hom	268.22	11	**atg-5**		UTR_3_PRIME: 128 bases from CDS				
I	1731010	A	T	SNP	Hom	117.15	4	tln-1, unc-35	talin	NON_SYNONYMOUS_CODING	N/I	aAc/aTc	0	+
I	1740691	*		INS	Hom	197.16	8	mppa-1	Mito Processing Peptidase Alpha	UTR_3_PRIME: 74 bases from CDS				+
I	1763076	G	T	SNP	Hom	195.35	6	lin-65	SynMuvB proline rich	NON_SYNONYMOUS_CODING	A/S	Gct/Tct	0	
I	1766320	*	+C	INS	Hom	356.8	10	lin-65		UTR_3_PRIME: 289 bases from CDS				
I	1771464	*		DEL	Hom	141.44	7	Y71G12B.25	protein_coding	UTR_3_PRIME: 80 bases from CDS				
I	1771511	*		INS	Hom	244.19	14	Y71G12B.25		UTR_3_PRIME: 33 bases from CDS				
I	1801557	C	A	SNP	Hom	142.91	6	Y71G12B.5	protein_coding	NON_SYNONYMOUS_CODING	P/T	Cct/Act	0	
I	1801727	A	G	SNP	Hom	415.92	12	Y71G12B.5		NON_SYNONYMOUS_CODING	S/G	Agt/Ggt	0	
I	1802798	A	G	SNP	Hom	183.84	7	Y71G12B.5		NON_SYNONYMOUS_CODING	I/V	Att/Gtt	0	
I	1803484	A	G	SNP	Hom	322.47	12	Y71G12B.5		NON_SYNONYMOUS_CODING	I/M	atA/atG	2	
I	1803485	A	G	SNP	Hom	322.47	12	Y71G12B.5		NON_SYNONYMOUS_CODING	M/V	Atg/Gtg	0	
I	1803997	G	T	SNP	Hom	272.24	8	Y71G12B.5		NON_SYNONYMOUS_CODING	R/L	cGg/cTg	0	

I	1804164	A	G	SNP	Hom	438.38	12	Y71G12B.5		NON_SYNONYMOUS_CODING	T/A	Acc/Gcc	0	
I	1811285	G	A	SNP	Hom	285.65	8	pghm-1	extented lifespan	NON_SYNONYMOUS_CODING	V/I	Gtc/Atc	0	
I	1823449	C	A	SNP	Hom	445.64	12	chaf-2	chromatin assembly	UTR_5_PRIME: 7 bases from TSS				+
I	1825924	*		DEL	Hom	347.6	11	Y71G12B.1		UTR_3_PRIME: 17 bases from CDS				+
I	1832531	G	A	SNP	Hom	401.01	11	C53H9.3	protein_coding	UTR_5_PRIME: 51 bases from TSS				
I	1841231	C	T	SNP	Hom	155.25	6	egl-30	G protein alpha subunit	UTR_3_PRIME: 341 bases from CDS				+
I	1841245	T	C	SNP	Hom	194.5	7	egl-30		UTR_3_PRIME: 355 bases from CDS				
I	1846453	T	C	SNP	Hom	167.29	5	tag-96	protein_coding	NON_SYNONYMOUS_CODING	F/S	tTc/tCc	0	+
I	1850251	C	G	SNP	Hom	350.74	10	tag-96	galactokinase	NON_SYNONYMOUS_CODING	D/E	gaC/gaG	1	
I	1850423	*		INS	Hom	151.46	8	tag-96		UTR_3_PRIME: 19 bases from CDS				
I	1874807	T	G	SNP	Hom	205.74	6	tub-2	Tubby-related	NON_SYNONYMOUS_CODING	V/G	gTt/gGt	0	+
I	1879747	A	G	SNP	Hom	194.91	6	tub-2		NON_SYNONYMOUS_CODING	T/A	Act/Gct	0	
I	1894681	C	T	SNP	Hom	144.86	6	Y71G12A.4	protein_coding	NON_SYNONYMOUS_CODING	G/E	gGg/gAg	0	
I	1910488	T	G	SNP	Hom	38.39	2	trpp-10	protein_coding	NON_SYNONYMOUS_CODING	F/L	ttT/ttG	1	+
I	1917998	A	C	SNP	Hom	482.79	13	trpp-10		NON_SYNONYMOUS_CODING	Q/H	caA/caC	1	
I	1941145	A	C	SNP	Hom	164.26	5	Y51F10.4	protein_coding	NON_SYNONYMOUS_CODING	T/P	Acg/Ccg	0	+
I	1941368	A	G	SNP	Hom	189.28	6	Y51F10.4		NON_SYNONYMOUS_CODING	E/G	gAa/gGa	0	
I	1945085	*		DEL	Hom	114.12	9	Y51F10.4		UTR_3_PRIME: 51 bases from CDS				
I	1950653	*		DEL	Hom	305.02	18	spe-48	ubiquitin-associated protein	UTR_3_PRIME: 122 bases from CDS				
I	1953467	T	C	SNP	Hom	228.43	7	spe-48		NON_SYNONYMOUS_CODING	K/E	Aaa/Gaa	0	
I	1958466	C	T	SNP	Hom	434.73	13	spe-48		UTR_5_PRIME: 10 bases from TSS				
